# Report of the Committee of Academy of Medicine on Ship Fever

**Published:** 1848-01

**Authors:** 


*Report of the Committee of Academy of Medicine on Ship Fever.*—We have been looking with some impatience for this promised report. When will it be forthcoming?

